# Impact of the COVID-19 pandemic on core surgical training

**DOI:** 10.1177/0036933020949217

**Published:** 2020-08-09

**Authors:** Khurram Shahzad Khan, Rona Keay, Morag McLellan, Sajid Mahmud

**Affiliations:** 1 ST6 in General Surgery, University Hospital Hairmyres, East Kilbride, Scotland, UK; 2 Clinical Development Fellow in Emergency Medicine, University Hospital Hairmyres, East Kilbride, Scotland, UK; 3 CT2 in General Surgery, University Hospital Hairmyres, East Kilbride, Scotland, UK; 4 Consultant Surgeon, University Hospital Hairmyres, East Kilbride, Scotland, UK

**Keywords:** Core surgical training, COVID-19, pandemic, competencies, surgical skills

## Abstract

**Background and aims:**

COVID-19 pandemic has caused significant disruption in training which is even more pronounced in the surgical specialties. We aim to assess the impact of COVID-19 pandemic on core surgical training.

**Methods:**

All core surgical and improving surgical trainees in West of Scotland region were invited to participate in an online voluntary anonymous survey via SurveyMonkey.

**Results:**

28 of 44 (63.6%) trainees responded, 15 (53.6%) were CT1/ST1. 14 (50.0%) working in teaching hospital and 15 (53.6%) working in general surgery. 20 (71.4%) felt that due to the pandemic they have less opportunity to operate as the primary surgeon. 21 (75.0%) have not attended any outpatient clinics. 8 (28.6%) did not have any form of access to the laparoscopic box-trainer. 20 (71.4%) felt their level of confidence in preforming surgical skills has been negatively impacted. 18 (64.3%) found it difficult to demonstrate progress in portfolio. 21 (75.0%) trainees have not attended any teaching. 10 (35.7%) trainees have been off-sick. 8 (28.6%) trainees have felt slightly or significantly more stressed.

**Conclusion:**

COVID-19 pandemic has an unprecedented negative impact on all aspects of core surgical training. The long term impact on the current cohort of trainees is yet to be seen.

## Introduction

Early surgical training in the United Kingdom consists of entry into one of two training programmes – Core Surgical Training (CST) or Improving Surgical Training (IST). Both programmes commence following the completion of foundation years of training. Given the practical nature of surgical specialties, an important part of training involves gaining experience in the operating theatre and endoscopy department. Skills in audit, teaching and research must also be shown. Improved proficiency must be demonstrated to allow completion and subsequent progression to Higher Specialty Training.^1–4^

There have always been barriers to providing high quality surgical training programmes in the United Kingdom. These include the use of trainees for provision of service, reduction in study budgets and stricter rules regarding working hours, as a result of the European Working Time Directive.^5^ The latest barrier to providing adequate surgical training is the Covid-19 pandemic.

Covid-19 was first recognised in Wuhan, China, in December 2019. Since then, it has spread worldwide to become a global pandemic and has been declared a public health emergency of international concern by the World Health Organisation (WHO).^6^ The pandemic is already having huge consequences globally, disrupting economies and international travel. In the UK, the aim has been to prevent the National Health Service (NHS) from becoming overwhelmed. As a result, most elective activity in all specialties has been cancelled. There has been redeployment of staff to areas where they are most likely to be necessary. In surgery, in response to guidance published in March by the Royal College of Surgeons of England, most elective operating lists have been cancelled, with only some urgent cancer cases proceeding. The aim has been for conservative, rather than surgical management where possible. This has been necessitated by the need for increased staffing levels and the space required to manage COVID-19 patients. The impact of performing non urgent procedures, particularly laparoscopic procedures, on the safety of staff has also been questioned, due to aerosol generation and potential virus transmission.^7,8^ Operating on patients with Covid-19 also appears to have poorer outcomes.^9^

The risk to health of staff is apparent. In Italy, up to 22% of health care staff are known to have been infected with Covid-19, and some have subsequently died. The stress of the threat to personal health, together with the mental and physical exhaustion of long shifts performed in often unfamiliar environments, also risks harming the mental wellbeing of healthcare staff.^7^

It is thought that the pandemic will have a significant impact on medical education, including surgical training.^10^ In Italy, COVID-19 has been shown to have a detrimental effect on the training of Urology registrars, with a significant reduction in clinical and surgical exposure.^11^ In the UK, normal training rotations have been suspended. There are few elective surgical theatre lists or clinics. Many educational events, including conferences and examinations, have been cancelled.^12^ The timing of the pandemic could have an effect of training progression, with trainees unable to achieve the competencies required to move on to the next stage of training.

Various methods of maintaining an education programme have been suggested, taking into account the reduction in practical training opportunities and the need to maintain social distancing. These include the use of technology for web-based learning and participation in surgical simulation. Studies highlight that these are no substitute for practical, hands on experience, but they may be used as a means of continued development in exceptional circumstances.^13,14^ In order to develop an appropriate education programme in response to the pandemic, it is essential to determine the requirements and difficulties our trainees are facing.

The aims of this study were firstly to assess the impact of the pandemic on core surgical training, including operative and endoscopic skills, and secondly, to determine the wellbeing of CST and IST.

## Material and methods

All Core Surgical Training (CT1 and CT2) and Improving Surgical Training (ST1 & ST2) in the West of Scotland region were invited to participate in an online voluntary anonymous survey. The survey was sent on 5th of April 2020, approximately six weeks after the WHO declared the pandemic, and was open for two weeks, before results were collected on the 19th of April 2020. The survey consisted of 51 questions, divided into nine sections. One reminder email was sent to recipients after the initial invitation.

Categories of the survey included trainee demographics and stage of training, operative and endoscopic experience, simulation training, general surgical activity, confidence, work pattern, academic activity, courses and conferences, exams and recruitment, and finally sickness and wellbeing.

The survey platform SurveyMonkey was used to distribute the survey and to collect responses. The majority of questions were multiple choice but also included a section for free text comments and feedback. Nominal data are presented as percentage of responses per category.

### Ethical considerations

A written permission was taken from the core surgery Training Program Director (TPD) to conduct the study. Trainees were initially contacted by the TPD to inform them of the survey and were given the opportunity to decline.

This was a voluntary anonymised survey; none of the questions in the survey were compulsory, hence the participants had the opportunity to skip questions if they wished to do so. The purpose and aims of the study were explained in an invitation email and participants provided a declaration of consent, giving permission for their anonymous results to be used in the study.

## Results

### Background

**Figure 1.**
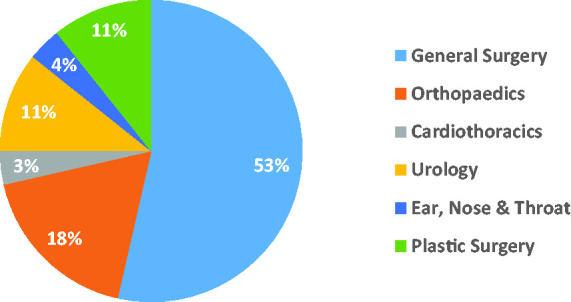
Surgical specialty in which the trainees are currently working.

Of the 44 trainees sent the online survey, 28 (63.6%) responded. 15 (53.6%) were CT1/ST1. 20 (71.4%) were 26 – 30 years old and 8 (28.6%) were 31 – 35 years old. 18 (64.3%) of the trainees lived with a partner or spouse during the pandemic. Half of the trainees (50.0%) were working in a district general hospital. Figure 1 shows the trainees’ specialties – 15 (53.6%) were working in general surgery.

### Operative & endoscopic experience and surgical clinic

**Figure 2.**
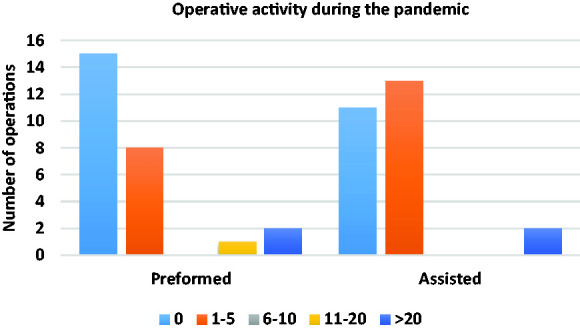
Number of operations performed or assisted in since the beginning of the pandemic.

Figure 2 shows the operative activity since the pandemic (for six weeks). 20 (71.4%) trainees felt that, due to the pandemic, they had less opportunity to operate as the primary surgeon. None of the trainees had performed any form of endoscopy during the pandemic. 21 (75.0%) trainees had not attended an outpatient clinic since the beginning of the pandemic. 3 (10.7%) trainees had attended a single clinic. One (3.6%) trainee attended 5 outpatients’ clinics. This trainee was working in orthopaedic surgery.

### Simulation training

Fourteen (50.0%) trainees had access to their own laparoscopic box trainer, 9 (32.1%) did not think they had adequate access to the laparoscopic box trainer and 8 (28.6%) did not have any access to the laparoscopic box trainer at all. 14 (50.0%) felt that a laparoscopic box trainer would help to maintain laparoscopic skills during the pandemic.

### General surgical activity

**Figure 3.**
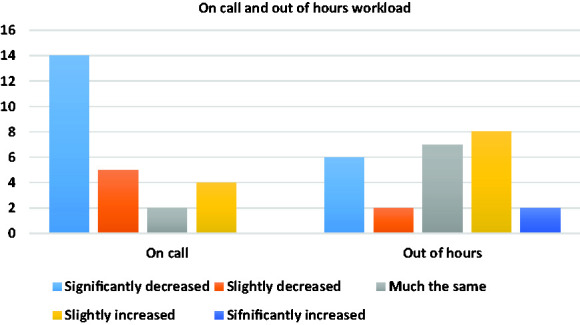
Change in workload since the pandemic during on calls and out of hours.

Figure 3 shows the change in workload during on calls and out of hours work. 19 (67.9%) of trainees experienced either a slight or a significant decrease in on call commitments during the pandemic. In contrast, 10 (35.7%) trainees had their out of hours commitment either slightly increased or significantly increased. 15 (53.6%) trainees had experienced either a slight or significant increase in their compulsory rest/off days during the pandemic.

### Confidence

**Figure 4.**
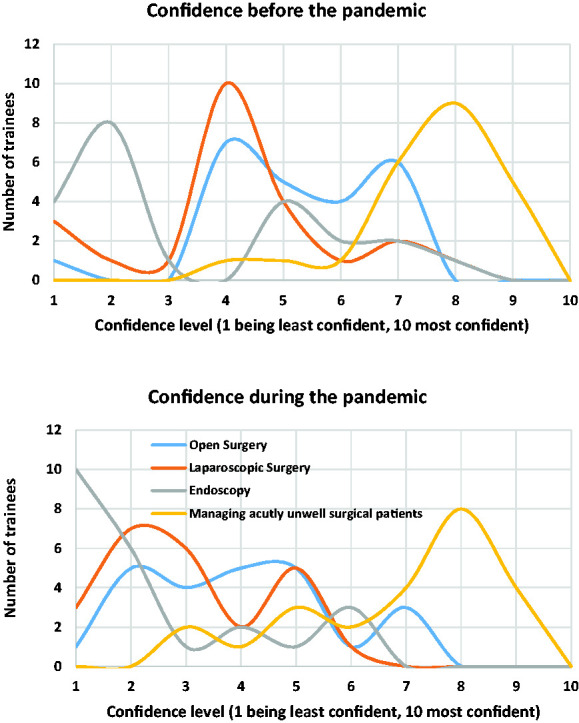
Compares the confidence of trainees before and during the pandemic in open and laparoscopic surgery, endoscopy and managing acutely unwell surgical patients.

Twenty (71.4%) trainees felt that their confidence performing surgical skills had been negatively impacted by the pandemic. Figure 4 compares confidence levels before and during the pandemic for open and laparoscopic surgery, endoscopy and managing acutely unwell patients.

### Work pattern

Nine (32.1%) trainees had been redeployed to another specialty during the pandemic. 8 (28.6%) had also taken on additional responsibilities, for example hospital at night cover and medical ward rounds.

### Academic activity

Eighteen (64.3%) trainees had found it difficult to demonstrate progress in their portfolio and 21 (75.0%) felt that there was less content to fill in Workplace Based Assessments (WBAs). 20 (71.4%) trainees felt there were fewer opportunities to undertake an audit. However, 10 (35.7%) trainees felt they had more time for audits.

According to 10 (35.7%) of the trainees, there was more time to focus on research projects, but many trainees experienced limited approvals for projects and reduced access to resources during the pandemic.

### Courses, conferences, exams and recruitment

Since the pandemic, 21 (75.0%) trainees had not attended any teaching. 3 (10.7%) attended teaching, all in orthopaedic surgery via Zoom software. 23 (82.1%) had their teaching cancelled. A further 7 (25.0%) had their teaching re-delivered by webinars. The development of new teaching material was by hospital or deanery in 90% of cases.

Twenty two (78.6%) of the trainees had their pre booked courses, conferences or exams cancelled due to the pandemic. All of the trainees were either reimbursed for the cost involved or have rebooked on a future date. Six (21.4%) trainees have been impacted by the changes in recruitment due to the pandemic.

### Sickness and wellbeing

Since the start of the COVID-19 pandemic 10 (35.7%) trainees have been off sick (including self-isolation). Of these, 9 were directly related to COVID-19. 10 (35.7%) trainees had their pre-planned annual leave cancelled.

Nine (32.1%) trainees felt that the pandemic had positively affected work-life balance whereas 17 (60.7%) had either experienced no change or felt it was negatively affected. 13 (46.4%) trainees spent more time with their family due to pandemic.

Eight (28.6%) trainees felt slightly or significantly more stressed as a result of the pandemic. 2 (7.1%) felt definitely burned out. 9 (32.1%) of the trainees were not sure what type of emotional and mental health support was available in their hospital. Eighteen (64.3%) trainees were either slightly or significantly more concerned about their career progression as a result of the pandemic.

## Discussion

There are multiple factors which have led to the disruption of early stage surgical training during the COVID-19 pandemic in the West of Scotland. These include a significant reduction in elective operating, the drive to manage emergency patients conservatively where possible, redeployment of staff to other clinical areas and centralisation of surgical services. Consultant-led operating together with restrictions to the numbers of staff allowed in theatres may also have resulted in reduced opportunities for junior trainees. The survey results clearly show that this has impacted on the confidence levels of trainees, particularly in laparoscopic surgery and endoscopy, but also in open surgery. Confidence levels have remained similar in the management of acutely unwell patients, which would be expected given continued emergency surgical presentations and the need to manage acute COVID-19 patients, particularly by redeployed staff. A minority of respondents had increased operating time which likely reflects benefit from the centralisation of services.

A crucial part of surgical training involves demonstrating progression. The survey has shown that trainees have found the completion of WBAs to be challenging. Opportunities to complete audit cycles have also been affected. A large proportion of trainees had had no formal teaching throughout the pandemic, although some specialties had provided ongoing teaching via online methods. Although allowances will be made for the impact of the COVID-19 pandemic at the Annual Review of Competency Progression (ARCP) and will likely not impact upon progression, within the context of the two year core training programme the consequences may be significant.

Trainee welfare was an issue with around one third of trainees off sick during the pandemic. A proportion also felt that their stress levels had risen and were concerned about career progression. Sickness absence and stress may also have compounded the lack of opportunity to gain practical experience and WBAs. Responders noted that the stress caused by COVID-19 has led to mental exhaustion and reduced motivation to focus on career progression at the present time, which is concerning. Many trainees were not aware of the support services available in their hospital. Hospital management and clinical supervisors have a crucial role in protecting the physical and mental wellbeing of trainees and should be aware of the resources available to all staff throughout the pandemic.

It is important that ways to continue to provide appropriate surgical training during the pandemic are explored. The use of technology could be crucial, with online platforms being a good option for providing teaching. This could be in the form of modules, lectures, or face to face using conference calling. Some trainees had access to their own laparoscopic simulators, but a proportion did not. The use of simulators could be an important tool for ongoing development of practical skills when real time operating is not possible, both in laparoscopic and open surgery as well as endoscopy. Some flexibility surrounding completion of portfolios may also be required.

Resumption of training activity and adaptation to the ongoing challenges is crucial, to minimise the impact upon training going forward. Acknowledging the impact of COVID-19 upon operative exposure and more generally upon traditional teaching methods, there is an urgency to finding solutions, particularly as disruption to practice continues. Increased use of technology, including webinars and online courses, offers a solution to maintain delivery of structured group teaching sessions and knowledge acquisition. Increasing access to simulation, such as utilizing the laparoscopic box simulator, could help to reduce the learning curve when approaching surgical procedure when subsequent opportunities arise. Ensuring that trainees are aware of academic opportunities that continue despite COVID-19, such as virtual conferences, could be important in addressing the perception that access to academia has decreased. However despite these strategies, the key is likely to be the development of an individualized approach, closely supported by trainers to identify shortcomings in trainees’ portfolios and maximize opportunities, whether progressing to CT2 or higher surgical training.

## Conclusion

It is impossible to fully assess the long term effects of the COVID-19 pandemic on the careers of early stage surgical trainees, but it is clear from the study presented in this paper that there has been at least a short term impact in the West of Scotland. These trainees are likely to be entering the next stage of training in August this year with decreased confidence levels and potentially decreased ability compared to their peers in previous years. At present, it is unclear how long social distancing measures and reduced hospital elective activity will be in place and, as such, further work will be valuable to determine how to best enhance surgical training during these unprecedented times.
